# Design and respondent selection of a population-based study on associations between breast cancer screening, lifestyle and quality of life

**DOI:** 10.1186/s12889-015-2603-7

**Published:** 2015-12-18

**Authors:** Tytti Sarkeala, Sirpa Heinävaara, Jonna Fredman, Satu Männistö, Riitta Luoto, Maija Jäntti, Nea Malila

**Affiliations:** Finnish Cancer Registry, Unioninkatu 20-22, 00130 Helsinki, Finland; UKK Institute, Kaupinpuistonkatu 1, 33500 Tampere, Finland; National Institute for Health and Welfare, Mannerheimintie 170, 00217 Helsinki, Finland

**Keywords:** Breast cancer, Screening, Quality of life, Lifestyle, Non-response, Bias

## Abstract

**Background:**

Only few studies have integrated breast cancer screening, lifestyle, and quality of life. Potential bias due to selective non-response may disrupt associations being investigated. We describe the design of a Finnish population-based study on associations between breast cancer screening and various indicators for lifestyle and quality of life, and evaluate the level of bias among the respondents from the first study rounds over 2 years.

**Methods:**

The study target population of 10 000, 49-year-old women was randomly drawn from the Finnish National Population Registry. The data included birth year, marital status, municipality, and primary language. Data on education were retrieved from Statistics Finland.

Questionnaires focusing on lifestyle-related risk factors and quality of life were sent to the target population in 2012–13, 1 year before the first invitation to organized breast cancer screening.

We evaluated associations between willingness to respond and demographic characteristics in the eligible study population. Additionally, we examined associations between the demographic characteristics and the Satisfaction With Life Scale (SWLS), and evaluated the impact of non-response using inverse probability weighting and multiple imputation.

**Results:**

The questionnaire response proportion was 52.4 %. Compared to non-respondents, respondents were more often married, academically educated, and native speakers of Finnish or Swedish. Nevertheless, the estimates of the SWLS among the respondents were in line with those corrected by non-response in the eligible study population.

**Conclusions:**

Based on the SWLS, the respondents are representative of women in the entire eligible study population.

## Background

Breast cancer is the leading cancer and most frequent cause of cancer death among women worldwide [1]. Screening is a major component of secondary breast cancer control. Cumulative evidence from randomised trials and observational studies have demonstrated screening to be effective in reducing breast cancer mortality among women aged 50–69 years [2–6].

Lifestyle is a major modulator of breast cancer risk, and changes in lifestyle have been shown to affect quality of life [7–10]. Both desirable and harmful lifestyle changes due to participation to screening have been reported from colorectal and lung cancer screening trials [11]. The results suggest that screening may induce desirable lifestyle changes but may also provide false reassurance to continue or to start unhealthy behaviour.

Previous studies on population-based breast cancer screening have concentrated on the screening process (participation, recall rate, false positive and false negative rate) and the outcome (mortality reduction) [12, 13]. Some have also investigated psychological distress due to false positive mammograms [14, 15]. No studies, so far, have assessed quality of life among the majority of the screened women, i.e. those receiving a normal or a false-negative screening finding. Furthermore, no studies have examined impacts of breast cancer screening on lifestyle.

In 2012, the Finnish Cancer Registry launched a population-based study to evaluate associations between breast cancer screening, lifestyle, and quality of life among middle-aged Finnish women. We report here the design of the study and assess the overall response, phases of response and the influence of non-response in associations being investigated, using demographic characteristics derived from the Finnish National Population Registry (FNPR) and the Statistics Finland (SF), and a Satisfaction With Life Scale (SWLS) addressed in the study questionnaire.

## Methods

The study target population of Finnish women born in 1963 (*n* = 5000) and in 1964 (*n* = 5000) was randomly drawn from the FNPR in 2012 and in 2013, respectively, using the year of birth as the only restricting factor. The study material including questionnaires with information letters and informed consent forms were mailed to the target population in 2012 and 2013, one year before the first invitation to organized breast cancer screening at the age of 50 years. The same study material will be mailed 1 year after the first screening invitation to the same women (in 2014 and 2015, respectively).

The study questionnaire focuses on perceived and lifestyle-related risk factors and lifestyle indicators, such as breast cancer in the family, concerns about breast cancer, hormone related factors, hormonal replacement, dietary habits, physical activity, obesity, and smoking. Factors relevant for mammography screening, such as screening experiences, screening outcome, and use of spontaneous breast cancer screening are also addressed.

**Fig. 1:**
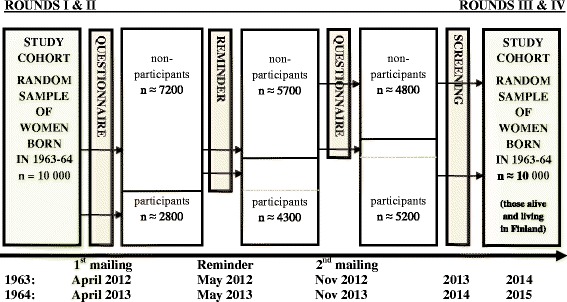
Study course and time frame for the birth cohorts 1963 and 1964 in 2012–15

The study has three phases in the mailing process. In the first and in the third phase, the eligible women (or the so far non-responding) receive all study material. In the second phase, they receive only a reminder letter (Fig. 1). The study participants are those returning both a filled questionnaire and a filled informed consent, others are non-participants.

Demographic characteristics for the whole study target population were obtained from the FNPR and the SF in 2012 and 2013. The FNPR data included year of birth, marital status, primary language, municipality, and data on children (birth year and sex). The SF data included information on education and occupation. Data on attendance, findings (true or false negative, true or false positive), breast cancer diagnoses, and deaths from breast cancer will be derived from the Finnish Mass Screening and Cancer Registries. The FNPR, the SF, the questionnaire, and the registry data can be linked using a social security number, which is unique to every person in Finland.

We calculated overall and phase-specific numbers of respondents and non-respondents, and the response rates among the study target population from the first study rounds over the years 2012 and 2013. For the number and rate of response, target population was followed from the date of the first mailing phase in 2012 and 2013 until January 31st the next year (in 2013 and 2014, respectively). Associations between non-response and the demographic characteristics were analysed using Poisson regression, and are reported by incidence rate ratios (IRR). Associations between the three various mailing phases and the demographic characteristics were analysed using ordinal logistic regression, and are reported by proportional odds ratios (POR). In each analysis, models without interaction terms were sufficient in describing the data.

The demographic characteristics applied in the analyses were the birth cohort (1963 or 1964), marital status, primary language, education, and university hospital region (based on information on municipality). Marital status was divided into the following categories: married (including also common-law marriages), single, divorced, and widow. Primary language was divided into categories Finnish, Swedish, and other, and education into categories primary (comprehensive education, 0–9 years), secondary (upper secondary general and/or vocational education, 9–12 years), and tertiary (higher and/or academic education, 12+ years). The five university hospital regions Helsinki (HYKS), Kuopio (KYS), Oulu (OYS), Tampere (TAYS) and Turku (TYKS) represented both geographical variation and density of the survey target population; Helsinki as the southern capital area (the most urban), Kuopio as the eastern area (mostly rural), Oulu as the northern area (mostly rural), Tampere as the central area (mostly urban), and Turku as the west-coast area (mostly urban). Those with unknown marital status as well as those living in the islands of Åland were excluded from the analyses due to small number of observations.

Potential bias due to non-response was addressed using the Satisfaction With Life Scale (SWLS) as an example. The SWLS is a five-statement, widely used generic instrument designed to measure cognitive judgements of satisfaction with one’s life [16]. The respondents are asked to indicate their agreement with each of the statements using 1 to 7 scale. The final score varies from 5 to 35 with seven categories, where the smallest category (5–9) describes those extremely dissatisfied, and the highest (31–35) those extremely satisfied with their lives.

The SWLS score was first analysed as a function of demographic characteristics among the study respondents using ordinal logistic regression. Thereafter, inverse probability weighting (IPW) and multiple imputation (MI) were employed to find out whether associations between the demographic characteristics and the SWLS score among the study respondents were similar to that of a corrected, complete data set, i.e. a data set with a hypothetical 100 % response rate [17]. In the IPW approach, the complete data set was generated by weighting the observed responses by the inverse of their predicted probabilities of being the observed response. In the MI approach, the SWLS estimates for the non-respondents were generated with a set of 50 imputations from the observed respondent data.

Since relationships between the demographic characteristics and the life satisfaction may vary between the respondents and the non-respondents (e.g. the married respondents may be more or less satisfied with their lives than the married non-respondents), alternative assumptions on the distribution of the SWLS in relation to marital status and education were generated. The observed marginal distribution of the SWLS among the respondents was impaired and improved by 4 % for the non-respondents, thus formulating two new SWLS scores for the corrected, complete data set. Thereafter, these new, overall SWLS scores were compared with the previously formulated scores.

Helsinki and Uusimaa Hospital District Ethics Committee has approved the study design (17.4.2012, 43/13/03/00/2012) and National Institute for Health and Welfare has given permission to perform the study and use the data (20.2.2014, THL/1697/5.05.00/2013).

## Results

The overall response rate in 2012–2013 was 52.4 % in the entire target population (*n* = 10 000), and 53.0 % in the eligible study population (*n* = 9894). The non-reachable members of the target population (*n* = 106) were not included in the eligible study population. These were women, who refused to participate, did not return the informed consent, could not be reached by mail, or had died during the study period.

The response rates after the first mailing phase were 27.3 % in the birth cohort 1963, and 29.1 % in the birth cohort 1964. The corresponding percentages after the second phase were 41.5 and 45.6 %, and after the third phase 51.8 and 53.0 %, respectively (Table 1).

**Table 1:** The intake of the 1963 and 1964 born study population in 2012–2013

	1963		1964		All	
	N	(%)	N	(%)	N	(%)
Study populaton	5000	(100)	5000	(100)	10,000	(100)
1st mailing	5000	(100)	5000	(100)	10,000	(100)
Respondents	1363	(27.3)	1454	(29.1)	2817	(28.2)
Declining	2	(0.0)	4	(0.1)	6	(0.1)
Reminder	3628	(72.6)	3536	(70.7)	7164	(71.6)
Respondents	713	(14.3)	823	(16.7)	1536	(15.4)
Declining	10	(0.2)	9	(0.2)	19	(0.2)
2nd mailing	2904	(58.1)	2692	(53.8)	5596	(56.0)
Respondents	520	(10.4)	375	(7.5)	895	(9.0)
Declining	22	(0.4)	5	(0.1)	27	(0.3)
No consent	12	(0.2)	19	(0.4)	31	(0.3)
Not reached	9	(0.2)	5	(0.1)	14	(0.1)
Deaths	6	(0.1)	3	(0.1)	9	(0.1)
Respondents	2596	(51.8)	2652	(53.0)	5248	(52.4)
Non-respondents	2343	(46.9)	2303	(46.1)	4646	(46.5)
Eligible subjects	4939	(98.8)	4955	(99.1)	9894	(98.9)

The distribution of demographic characteristics among the respondents, the non-respondents, and among the eligible study population is presented in Table 2. Compared to the non-respondents, the respondents were more often married, highly educated, and native speakers of Finnish or Swedish. The geographical distribution as well as the distribution by the birth cohort was similar among the respondents and the non-respondents. There were differences in the accumulation of respondents between the two birth cohorts over the three mailing phases in 2012 and 2013 (POR 1.08, 95 % CI 1.00–1.17) (Table 3). Nevertheless, the overall number of respondents as well as the distribution of demographic characteristics was similar in both birth cohorts (IRR 1.02, 95 % CI 0.97–1.08).

**Table 2:** Distribution of demographic characteristics among the eligible study population by the status of participation

	Respondents		Non-respondents		All	
	n	(%)	n	(%)	n	(%)
Marital status						
Married	3166	(60.3)	2533	(54.5)	5699	(57.6)
Single	1000	(19.1)	1135	(24.4)	2135	(21.6)
Divorced	975	(18.6)	877	(18.9)	1852	(18.7)
Widow	77	(1.5)	66	(1.4)	143	(1.5)
NA	30	(0.6)	35	(0.8)	65	(0.7)
Education						
Tertiary	2663	(50.7)	2031	(43.7)	4694	(47.4)
Secondary	2176	(41.5)	1958	(42.1)	4134	(41.8)
Primary	409	(7.8)	657	(14.1)	1066	(10.8)
Region						
HYKS	1853	(35.3)	1727	(37.2)	3580	(36.2)
KYS	775	(14.8)	701	(15.1)	1476	(14.9)
OYS	704	(13.4)	569	(12.3)	1273	(12.9)
TAYS	1202	(22.9)	1037	(22.3)	2239	(22.6)
TYKS	689	(13.1)	578	(12.4)	1267	(12.8)
Åland	25	(0.6)	34	(0.7)	59	(0.6)
Birth cohort						
1963	2596	(49.5)	2343	(50.4)	4939	(49.9)
1964	2652	(50.5)	2303	(49.6)	4955	(50.1)
Primary language						
Finnish	4789	(91.3)	4144	(89.2)	8933	(90.3)
Swedish	251	(4.8)	215	(4.6)	466	(4.7)
Other	208	(4.0)	287	(6.2)	495	(5.0)
All	5248	(100.0)	4646	(100.0)	9894	(100.0)

**Table 3:** Response rates, and demographic factors associated with the response and the phases of response. For the response, incidence rate ratios (IRRs) are shown, and for the phases of response, proportional odds ratios (PORs) are shown, both with 95 % confidence intervals (lower, upper)

Response	Response rate (%)	IRR	(Lower, upper)	POR	(Lower, upper)
Marital status					
Married	55.6	1.00	.	1.00	.
Single	46.8	0.85	(0.79, 0.92)	0.73	(0.66, 0.80)
Divorced	52.7	0.97	(0.90, 1.04)	0.94	(0.85, 1.04)
Widow	53.9	1.00	(0.80, 1.25)	0.87	(0.64, 1.18)
Education					
Tertiary	56.7	1.00	.	1.00	.
Secondary	52.6	0.93	(0.88, 0.99)	0.84	(0.77, 0.91)
Primary	38.4	0.69	(0.62, 0.77)	0.48	(0.42, 0.55)
Region					
HYKS	51.8	1.00	.	1.00	.
KYS	52.5	1.00	(0.92, 1.09)	1.01	(0.90, 1.13)
OYS	55.3	1.05	(0.96, 1.15)	1.07	(0.95, 1.21)
TAYS	53.7	1.02	(0.95, 1.10)	1.06	(0.96, 1.17)
TYKS	54.4	1.04	(0.95, 1.14)	1.10	(0.98, 1.24)
Birth cohort					
1963	52.6	1.00	.	1.00	.
1964	53.5	1.02	(0.97, 1.08)	1.08	(1.00, 1.17)
Primary language					
Finnish	53.6	1.00	.	1.00	.
Swedish	53.9	1.02	(0.89, 1.16)	1.00	(0.83, 1.20)
Other	42.0	0.84	(0.72, 0.98)	0.67	(0.55, 0.81)

**Fig. 2:**
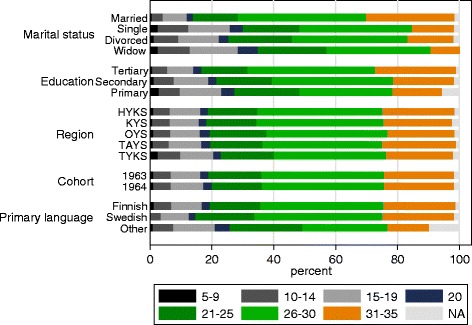
Distribution of Satisfaction With Life Scale (SWLS) by demographic characteristics among the study respondents. The final score of the SWLS varies from 5 to 35 with seven categories, where the smallest category (5–9) describes those extremely dissatisfied, and the highest (31–35) those extremely satisfied with their lives

Most of the study respondents were satisfied to their lives (Fig. 2). Associations between the demographic characteristics and the SWLS among the survey respondents and in the two corrected data sets are presented in Table 4 by PORs and 95 % CIs. The results show that the PORs of life satisfaction within each demographic category are similar among the study respondents and among both of the completed data sets. The reference categories “married”, “tertiary education”, “Helsinki region (HYKS)”, “the birth cohort 1963”, and “Finnish language” represent those most satisfied.

**Table 4:** Proportional odds ratios (PORs) with 95 % confidence intervals of the SWLS for various data. These data consist of the respondents and two corrected, complete data sets. The complete data sets were generated from the respondent data using inverse probability weighting (data^a^) and multiple imputation (data^b^)

	Respondents		Corrected, complete data^a^		Corrected, complete data^b^	
Response	POR	(lower, upper)	POR	(lower, upper)	POR	(lower, upper)
Marital status						
Married	1.00	.	1.00	.	1.00	.
Single	0.39	(0.34, 0.45)	0.39	(0.34, 0.45)	0.39	(0.33, 0.44)
Divorced	0.48	(0.42, 0.54)	0.47	(0.42, 0.54)	0.48	(0.42, 0.55)
Widow	0.33	(0.22, 0.50)	0.34	(0.23, 0.51)	0.34	(0.22, 0.51)
Education						
Tertiary	1.00	.	1.00	.	1.00	.
Secondary	0.76	(0.69, 0.85)	0.76	(0.68, 0.84)	0.76	(0.69, 0.85)
Primary	0.60	(0.49, 0.73)	0.60	(0.49, 0.74)	0.59	(0.48, 0.71)
Region						
HYKS	1.00	.	1.00	.	1.00	.
KYS	0.90	(0.77, 1.05)	0.91	(0.78, 1.06)	0.90	(0.77, 1.05)
OYS	0.84	(0.72, 0.99)	0.86	(0.73, 1.01)	0.84	(0.73, 0.97)
TAYS	0.93	(0.82, 1.07)	0.95	(0.83, 1.09)	0.94	(0.83, 1.06)
TYKS	0.79	(0.67, 0.93)	0.80	(0.68, 0.95)	0.78	(0.67, 0.95)
Birth cohort						
1963	1.00	.	1.00	.	1.00	.
1964	0.98	(0.88, 1.08)	0.97	(0.87, 1.07)	0.98	(0.89, 1.07)
Primary language						
Finnish	1.00	.	1.00	.	1.00	.
Swedish	0.94	(0.74, 1.20)	0.95	(0.75, 1.21)	0.94	(0.73, 1.20)
Other	0.51	(0.38, 0.68)	0.55	(0.41, 0.74)	0.53	(0.40, 0.69)

Improving or impairing the life satisfaction (SWLS score) in relation to marital status or education among the non-respondents did not change associations between the SWLS and the demographic characteristics among the respondents and the corrected data sets (data not shown).

## Discussion

We present a design of a Finnish study, which evaluates impacts of breast cancer screening on self-reported lifestyle and quality of life. We also report response rates, analyse the distribution of demographic characteristics over the respondents and non-respondents from the first, two study rounds, and evaluate the impact of non-response on the Satisfaction With Life Scale (SWLS) as an example.

The Finnish study is conducted during the years 2012–2015 among 10 000, randomly selected women born in 1963 and 1964 by sending them a questionnaire 1 year before and 1 year after their first invitation to organised breast cancer screening. After 2015, lifestyle and quality of life among the study respondents will be examined in relation to screening participation and results.

The first two rounds of the study were carried out in 2012 and 2013. During these years, the overall response rate among the target population was 52.4 %. Modest response rates have been reported also from other European studies [18–21]. Empirical assessments over the past decade have, however, shown that response rates may not be as strongly associated with the quality or representativeness of the study as has been believed. Even non-direct relationships between the response rate and the non-response bias have been reported [22]. It thus seems that the degree to which sampled respondents differ from the eligible survey population as a whole is central to evaluate the representativeness. Therefore, a study with a relatively high response rate may produce more biased results than a study with a lower response rate from a truly random and representative group of respondents [23–25].

Our study included three mailing phases within both rounds in 2012–2013. During the first and the third phase, the eligible population (or the so far non-respondents) received a questionnaire with an information letter and an informed consent. During the second phase, the eligible population received only a reminder. The additional mailing phases increased the overall response rate from 28.2 to 52.4 %. The accumulation of data differed between the study years (and birth cohorts). This did not, however, affect the distribution of demographic characteristics between the respondents and non-respondents. Additional contacts with reminders have been successful in increasing the sample size also in other studies [26]. Nevertheless, criticism has been given on inflating costs due to re-contacts as well as on the low impact of re-contacts on the response rate and data quality especially after the second contact [27].

Compared to the non-respondents, the respondents of our study were more often married, highly educated, and native speakers. This is in line with several previous studies, which have reported elderly, married, and educated women to be the most frequent respondents in health care studies [19, 21, 28, 29]. Despite these differences, the addressed quality of life estimate (the Satisfaction With Life Scale, SWLS) was similar among the respondents and in the complemented data sets constructed by the inverse probability weighting (IPW) and multiple imputation (MI). Moreover, improving or impairing the SWLS score in relation to marital status or education among the non-respondents did not change the overall distribution of the SWLS in the corrected data.

Adopting a comprehensive strategy to investigate missing data early in the research process gives researchers information necessary to evaluate key assumptions. Both the IPW and the MI methods are widely used to assess or improve the accuracy of results in various study designs [17]. In Finland, the IPW method has previously been applied e.g. to improve accuracy of results of a population survey using sociodemographic register data covering the whole study sample [30]. In the United States, the IPW and the MI methods have been utilized also to examine internal validity of estimates derived from longitudinal studies [31, 32].

## Conclusions

 Our results indicate that the estimates of the SWLS scores among the study respondents are similar to those corrected for non-response among the eligible study population (i.e. in the data sets complemented by the IPW and the MI methods). This may be due to the fact that the non-married, less-educated, and foreign speaking women formulated only a minority of the eligible survey population. It is also possible, that the demographic characteristics alone are not able to adequately address the wellbeing of the target population. Given these warranties and based on the SWLS estimate, the respondents of this population-based study may well represent the eligible study population and the female age cohorts 1963 and 1964 in Finland.
